# The role of traditional health practitioners in Rural KwaZulu-Natal, South Africa: generic or mode specific?

**DOI:** 10.1186/s12906-016-1293-8

**Published:** 2016-08-22

**Authors:** Thembelihle Zuma, Daniel Wight, Tamsen Rochat, Mosa Moshabela

**Affiliations:** 1 Africa Centre for Population Health, University of KwaZulu-Natal, Mtubatuba, South Africa; 2 MRC/CSO Social and Public Health Sciences Unit, University of Glasgow, Glasgow, Scotland, United Kingdom; 3 Human Sciences Research Council/Human and Social Development (HSD), Africa Centre for Population Health, University of KwaZulu-Natal, Mtubatuba, South Africa; 4 Developmental Pathways to Health Research Unit, School of Clinical Medicine, University of Witwatersrand, Johannesburg, South Africa; 5 School of Nursing and Public Health, University of KwaZulu-Natal, Durban, South Africa

**Keywords:** Traditional health practitioners, Traditional healing roles, Traditional health care, KwaZulu-Natal, South Africa

## Abstract

**Background:**

Traditional health practitioners (THPs) play a vital role in the health care of the majority of the South African population and elsewhere on the African continent. However, many studies have challenged the role of THPs in health care. Concerns raised in the literature include the rationale, safety and effectiveness of traditional health practices and methods, as well as what informs them. This paper explores the processes followed in becoming a traditional healer and how these processes are related to THP roles.

**Methods:**

A qualitative research design was adopted, using four repeat group discussions with nine THPs, as part of a larger qualitative study conducted within the HIV Treatment as Prevention trial in rural South Africa. THPs were sampled through the local THP association and snowballing techniques. Data collection approaches included photo-voice and community walks. The role identity theory and content analysis were used to explore the data following transcription and translation.

**Results:**

In the context of rural Northern KwaZulu-Natal, three types of THPs were identified: 1) *Isangoma* (diviner); 2) *Inyanga* (one who focuses on traditional medical remedies) and 3) *Umthandazi* (faith healer). Findings revealed that THPs are called by ancestors to become healers and/or go through an intensive process of learning about traditional medicines including plant, animal or mineral substances to provide health care. Some THPs identified themselves primarily as one type of healer, while most occupied multiple healing categories, that is, they practiced across different healing types. Our study also demonstrates that THPs fulfil roles that are not specific to the type of healer they are, these include services that go beyond the uses of herbs for physical illnesses or divination.

**Conclusions:**

THPs serve roles which include, but are not limited to, being custodians of traditional African religion and customs, educators about culture, counsellors, mediators and spiritual protectors. THPs’ mode specific roles are influenced by the processes by which they become healers. However, whichever type of healer they identified as, most THPs used similar, generic methods and practices to focus on the physical, spiritual, cultural, psychological, emotional and social elements of illness.

## Background

Literature suggests that traditional health practitioners (THPs) play a vital role in the health care of the majority of the South African population and elsewhere on the African continent [1, 2]. A THP is defined by the World Health Organisation (WHO) as a “a person who is recognised by the community where he or she lives as someone competent to provide health care by using plant, animal and mineral substances and other methods based on social, cultural and religious practices” [3]. The approach of THPs to health care is based on indigenous knowledge and belief systems [4, 5]. According to the South African Traditional Health Practitioners Act, THPs are consulted for their explicit linkage of health with patients’ social and cultural beliefs [6, 7].

Pretorius estimated that there were 150 000 to 200 000 traditional healers in South Africa [8]. In 2007, Gqaleni confirmed these estimates, indicating that there were about 25 000 THPs in KwaZulu-Natal (KZN). Only about 7000 were registered with their interim professional body [9]. In 2010, Ross estimated the ratio of THP to medical doctors asserting that there were about 250 000 to 400 000 THPs and 28 000 medical doctors in South Africa. However, it is important to note that these estimates were based on media reports. Ross [10] further pointed out that eight in ten black South Africans are believed to utilise THPs alone or along with Western medicine. Other studies suggest that about 70 % of black people in South Africa use THPs in one way or another [11–13]. There is general consensus that THPs are common and in demand within the patient population.

Traditional healing consists of a combination of healing practices such as divining, herbalism and spiritualism [14, 15]. THPs who are registered under the South African Traditional Health Practitioners Act include herbalists (*izinyanga* or *amaxhwele*), diviners (*izangoma*, *umthandazi* or *amagqirha*), traditional surgeons (*iingcibi*) who mainly do circumcisions, and traditional birth attendants (*ababelethisi* or *abazalisi*) [6]. However, presently it is difficult to determine the actual number of THPs, as many of them have remained unregistered, therefore a possible under estimate of the actual numbers of traditional healers exists [9]. Studies on THP utilisation have largely been within different tribal groups, distinguishing the differences and commonalities that seem to exist across different South African tribes [14, 16, 17].

While research studies show that the use of THPs has been a long standing component of health care practice in South Africa which contributes to the primary health care needs of people, it was only after 1994 that the South African government started to look at the prospect and feasibility of formally integrating traditional health care into the public health system [18]. More than two decades later, debates on how to go about executing the integration are still ongoing [1, 12, 19]. Attempts to create accreditation for THPs in the public health system have not yet succeeded [20, 21]. Concerns raised in the literature include the current lack of clarity as to whether or not THPs can be embraced by health practitioners whose philosophies of health care are based on the modern, biomedical approach [5]. A further challenge remains the lack of evidence for THPs diagnostic procedures, methods and training [20]. Unfortunately, the processes involved in traditional healing do not lend themselves to evaluation and measurement, and precise data, using both qualitative and quantitative methods, are still lacking [22].

Studies report that the knowledge applied by different types of healers is a product of, and is dependent on, the different disciplines of traditional healing to suit different aims and functions [23]. However, evidence documenting how THP’s roles are related to their categories is scant [16, 24]. Existing evidence focuses primarily on how THPs are initiated into the role of healing, their socio-cultural profile and traditional healing practices and methods [16, 22, 24]. However, further research is needed to understand the cultural specificity of THP types, how they vary, and how they are related to their roles.

In sub-Saharan Africa, THPs’ roles are constantly met with positive and negative criticism [25]. As a result, THPs in South Africa have had to fight for recognition by different quarters of society, including the Department of Health, Health Professions Council of South Africa (HPCSA), medical aid schemes and other authorities concerned with health care [5, 26]. For example, the Traditional Health Practitioners Act (No. 35 of 2004) does not mention religion, initiation, spirits, mediums, possession or trance states, all of which are associated with traditional healing in the popular and academic literature [27]. Having excluded most of what traditional healers are commonly understood to do, the Act ignores the importance of their role in spiritual enhancement when treating people, in addition to focusing on the body. A further example is that THPs have been excluded under the Health Professions Act of 1974, which is not the case for other health care practitioners in South Africa [23].

Gaps identified in literature around policy and accreditation for THPs in the public health system are largely due to the lack of systematic evidence about their healing roles, practices and methods. The current study will, therefore, describe the roles, methods and practices of individuals involved in traditional healing among the Zulu tribal group in a rural area in Northern KwaZulu-Natal province, South Africa. To achieve this aim, the study will explore the processes that are followed in becoming a traditional healer and how these processes are related to THP roles. The study seeks to understand how the indigenous methods and practices involved in traditional healing are related to THP identity.

## Methods

### Study design

A qualitative research approach was best suited to this study as the focus was on understanding subjective experiences and knowledge of THPs. The study was exploratory and descriptive in nature.

### Study setting

The study used qualitative data collected as part of a larger Treatment as Prevention (TasP) trial. The TasP trial is a cluster-randomized trial coordinated by an international study group at the Africa Centre for Population Health (REF: BFC 104/11). The trial has implemented universal and repeat home-based HIV-testing of all resident adults as standard of care and immediate ART initiation is implemented as the intervention [28]. A qualitative sub-study was conducted between January 2013 and July 2014, as part of the TasP trial [29]. The qualitative study explored among community members, including THPs, access to health care, how HIV/AIDS is managed and approached, as well as local practices that facilitate or hinder HIV testing, ART initiation and adherence [29]. Group discussions were conducted to explore perceptions of community members over time.

The area in which the study was conducted forms part of the Hlabisa health sub-district, one of the five sub-districts in the rural district of UMkhanyakude in Northern KwaZulu-Natal, South Africa. Adult unemployment in UMkhanyakude district is 67 %, and only 10 % of households are within 15 min’ travel time (driving) of a health clinic [25]. Approximately 76.8 % of the population in the sub-district are classified as residing in a rural area and approximately 92 % of the population speak isiZulu as a first language [30].

### Sampling

For this study we sought THPs who resided within the TasP trial community, were 16 years old and above, were willing to participate and commit to the 18 month period the study was scheduled for, and if they were willing to provide informed consent. Two sampling techniques were used: purposive and snowball sampling [31, 32]. Four THPs were purposefully sampled using a list of contact details provided by the local association of THPs, and obtained from the Africa Centre Community Engagement Unit (CEU) [33].

The community engagement unit (CEU) of the Africa Centre keeps the community informed on the research studies carried out by the Africa Centre. The CEU, serves as an entry point into the community, and supports the research conducted by the Centre by introducing different studies and educating local communities. Education provided to community members includes information on HIV/AIDS, particularly HIV testing and treatment, TB, and prevention of mother to child transmission of HIV (PMTCT). Further, the CEU keeps an open dialogue with community members in order to safeguard their voice in research processes and assist in ensuring that the research conduct complies with human rights and ethical standards. Community education is conducted through regular roadshows, radio broadcasts, focused community dialogues and press coverages. The contact list from CEU was requested from the chairperson of the traditional healer’s association in Hlabisa. The list included seven names of THPs who resided in the TasP trial community. However, only the first four were contacted and included in the study in order to allow for the snowballing of additional THPs not necessarily registered with the healer’s association.

The first four successfully contacted THPs were asked to meet with the researcher in the community. In the meeting, the researcher explained key issues about the study such as who was eligible to participate in group discussions, and requested them to introduce other THPs in the community through a snowballing technique. The researcher contacted a further five likely participants by phone to introduce and explain key issues of the study. All nine THPs approached, verbally consented to participate during initial contact. Among the contacted THPs, there was no refusal to participate. With the help of *Induna* (traditional headmen), a venue was secured where four group discussions with THPs were conducted. At the beginning of the first group discussion, the researcher went through study information, confidentiality and informed consent and each participant signed individual consent prior to the start of the first group discussion.

### Data collection

Group discussions were conducted between February and November 2013 in isiZulu, the first language of the participants and the facilitator (TZ). This qualitative study investigated the wider context in which the TasP trial was being conducted and involved THPs directly participating and not participating in the clinical component of the trial. Group discussion guides were initially designed in English and later translated to IsiZulu. The Community Advisory Board (CAB) and CEU of the Africa Centre confirmed group discussion guides for language appropriateness before they were adopted in the study.

**Fig. 1:**
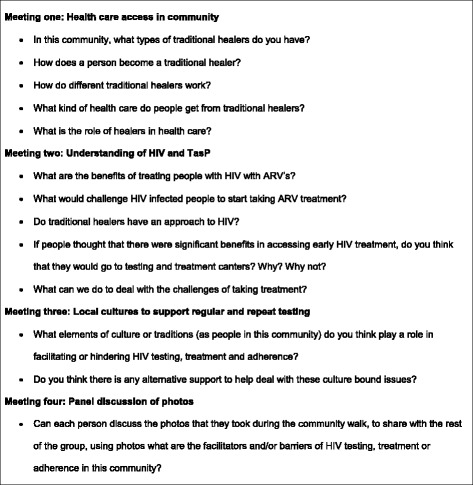
Topic guide for group discussions with THPs

Discussions were conducted in a community church hall. Discussions took between 60 and 120 min, and at the end of each discussion THPs were provided with lunch and transport reimbursement of ZAR 50. All group discussions were conducted by the same facilitator and the first author of this paper (TZ), who is also a first language Zulu speaker. Each of the four repeat group discussions were dedicated to a broader topic, but not mutually exclusive. Table 1 presents an overview of broad topics discussed over repeat group discussions as well as participants’ attendance. Figure 1 illustrates broad questions used to facilitate group discussions.

**Table 1:** Participants’ attendance over repeat group discussions, topics discussed and methods used per meeting

	Time point 1	Time point 2	Time point 3	Time point 4	Time point 5
	FGD 1	FGD 2	FGD 3	Community walk/taking of photos	FGD 4
Topics covered:	Understanding THPs: who are THPs, how do they become, what are their practices	THPs approach to illnesses including HIV/AIDS: How do they manage illness, do they refer patients to Western biomedical practitioners, when do they refer.	Local traditional practices that support or prevent HIV testing, initiation and retention in HIV/AIDS care	Community Walk and taking of photos representing facilitators and barriers to HIV testing, treatment initiation and adherence	Group discussion based on the photos taken in time point 4 about facilitators and barriers to HIV testing, treatment initiation and adherence
Dates	22/02/2013	30/05/2013	31/07/2013	09/10/2013	13/11/2013
Attendance	9 attended/0 absent	9 attended/0 absent	8 attended/1 absent	8 attended, 1 absent	7 attended, 2 absent
Method used to gather data	Individual and group narrative	Individual and group narrative	Individual and group narrative	Community walk; taking of photos	Individual and group narrative

Group discussion transcripts were translated into English by two trained translators and validated by TZ. Through the course of the study, three participants withdrew from group discussions and reported that they were undertaking traditional healing work away from their communities.

### Data analysis and interpretation

The study used content analysis to examine the role identity of THPs [34, 35]. Role identity theory was used to understand how THPs define and give meaning to themselves in relation to their traditional healing role. Using role identity, we specifically explored the data to explain how THP’s roles develop and what influences their practices. Role identity is defined by Farmer et al. as a process through which individuals give meaning to themselves in relation to a specific role [36]. The framework is used to explain and explore how roles develop and how they are represented by people in their work environment or in other social contexts [37]. Group discussion transcripts were coded manually using a coding framework that was developed from both deductive and inductive categories in two steps [38]. First, it was developed to focus on specific broad areas contained in the group discussion guides, and, second, codes were generated from the narratives of THPs. Categories were reviewed by four researchers (TZ, DW, TR and MM) for redundancy, and similar codes and categories were grouped under a single higher order category. This process was repeated until all transcripts were reviewed and the codebook had reached saturation with no new categories and content codes emerging. Table 2 shows an example of the coding framework.

**Table 2:** A Coding framework for the findings relating to the study of THPs roles

Category	Description	Content codes	Examples from transcripts
Typology	Type of healing practitioner	*ISangoma*_associated to ancestral spirits, is a diviner	*1.“……All the ones I have mentioned are all Sangomas. There are many of them, I have just mentioned a few of them” (Male, Interview 3)*
		*INyanga* _acquires knowledge of healing using plants, herbs and animal parts, is a herbalist	
			*2. “…..there is someone that I know who is a Sangoma, and we are coming from the same family. She also works both as Umthandazi and Inyanga like me” (Female, FGD1, Traditional healer)*
		*UMthandazi* _associated to religion & ancestral spirits, is a spiritualist/faith healer	
Calling Initiation Training	Process or journey of being appointed to become a traditional healer which includes training & completion (graduation). The process is characterised by performing different activities and rituals	Spiritual	*1.“This Spirit just comes to you unexpectedly. It comes as a messenger, the Holy Spirit just comes down to you and you will not have an idea of what happened to you” (Female, FGD1, Traditional healer)*
		Ancestral	
		Personal Choice	
		Refusal/Consequences	
		Training Institution	
		Apprenticeship	
		Rituals	
		Graduation	
Healing Practice	Process that a healer & patient go through to identify, explain, discuss & negotiate appropriate treatment with patient.	Causation	*1.“ The patient told me that umndawe [spirit] is high and he needs to start doing amagobongo [traditional ritual]. He was giving me an instruction as a healer! I am the one who is supposed to know what is wrong with him, and what help he needs, I am not supposed to take instructions from him. (Female, FGD 3, Traditional healers)*
		Source	
		Co-production	
		Assigning	
		Prescribing	
		Preparing	

Data that were relevant to each category and content code were collated according to the identified categories and extracted into an excel spreadsheet. Initial themes were then generated. Themes were refined and validated by two researchers (TZ and MM). Themes were more clearly delineated during write up. For this paper, themes clarifying the processes followed in becoming a THP, how the processes are related to THPs roles and THP’s identity were extracted. Direct quotes from THPs are used to illustrate the findings, indicated in italics. Each THP was allocated a unique identifying code and quotations are presented below together with three important participant characteristics (Gender; focus group time point; and type of healer) listed alongside each quote, for example: (Female, FGD1, *Isangoma*). Within quotations, text which is placed inside brackets () translates Zulu words, while text inside square brackets [] illustrates TZ’s (a Zulu speaker) explanations of phrases.

### Ethics permission

Ethical approval for the first phase of the TasP trial (which includes the social science sub-studies) was obtained from the University of KwaZulu-Natal Biomedical Research Ethics Committee (BREC) in 2011 (REF: BFC 104/11). A further approval was sought for the full protocol developed for the social science sub-studies, separate to the approval granted for the trial in 2012 (REF: BE090/12). Ethical clearance for this study was granted by BREC in 2015 (REF: BE432/15). THPs were asked for consent to audio record group discussions. No real names of THPs were used in this study as a reflection of best practice social science reporting [39].

## Results

### Profile of participants

The group discussions included nine THPs, most of whom were women (7/9). All the women were aged over 40, while the two men included in the sample were younger (<35 years). Three female THPs practised as *Isangoma/Inyanga* (diviner/herbalist). The three had started traditional healing as *Isangoma* (diviner) and later also trained as *Inyanga* (herbalist). Two THPs, one male and one female practiced as *Umthandazi/Inyanga* (faith healer/herbalist). They had both started traditional healing as *Umthand*azi (faith healer) and later also trained as *Inyanga* (herbalist). One male THP practiced as *Isangoma/Umthandazi* (diviner/faith healer). Two female THPs practiced as *Isangoma* (diviner), and another practiced as *Inyanga* (herbalist).

The findings are presented as a synthesis of narratives from THPs. The analysis identified a number of critical features describing the calling to become a THP, the typology and role of THPs and THP’s ancillary roles.

### The calling to become a traditional healer

THPs used the generic term ‘*umelaphi wendabuko’* (traditional healer) to refer to a person they recognised to provide traditional health care in their community. Within this generic category, mode specific terms based on a THP’s calling were used to help identify different categories of healers. *Isangoma* (diviner) and *Umthandazi* (faith healer) referred to a person who had been called by their *Amadlozi* (ancestors) or *Isithunywa* (God-sent messenger) to become a healer. A person who had gone through an intensive process of learning about traditional medicines and practices was referred to as *Inyanga* (herbalist). It was reported that the type of healer one became was not self-determined but instead was determined by spiritual entities or *Amadlozi* (ancestors) and required demanding and difficult training which participants had no choice but to comply with. Not all participants agreed that the calling was a spiritual experience. Some indicated that some people experienced illness as a consequence of the calling.

*“What I was trying to explain to you is that we do not call for this. It is something that you just get and you don’t invite it. It is like an illness, you don’t invite it”. (Female, FGD1, Isangoma/Inyanga)*

In the course of group discussions, the same participant restated the fact that as THPs, they do not have a choice but that they are given the healing gift by their ancestors and have a responsibility to follow it. Thus, once the calling is bestowed upon a person, the role of becoming a THP has to be fulfilled.

*“…..it comes to you uninvited it will come like a disease you can’t refuse” (Female, FGD1, Isangoma/Inyanga)*

Participants were in agreement that when an individual ignored, misunderstood, denied or refused the calling, *Amadlozi* (ancestors) brought misfortune or illness to that individual. The consequence or punishment of refusal was described as on-going physical, mental, psychological illness and social instability.

*“…. I stopped school because in the course of the year, I will perform well and do like other learners. But when we were about to write exams, I would not hear the teacher and I would just feel like leaving the class and just wander around. I would not disturb other learners at school…. (Male, FGD1, Traditional healer)*

Often the calling manifested in individuals as early as adolescence. One healer said that even though he recognised he was formally called into traditional healing when he was an adolescent, he believed that the calling was inevitable, and had been present all his life, that it was something that he was born with. His belief was that it already existed in him before he could discern it was there, therefore it was a part of him from birth.

*“It is something that I was born with. When I started going to the Zionist church, I already had this thing (ancestral spirit) in me” (Male, FGD1, Isangoma/Inyanga)*

It was stated that often family members, parents in particular, did not accept their child being called by ancestors to become a THP for the fear of their child being burdened by the responsibility that comes with such work. The gruelling process of becoming a healer was undesirable to families, mainly because people did not comprehend it.

*“When I started speaking in tongues because of the spirits that I had in me, my family refused to believe it. Most families do not accept such a thing when it happens to their own family members, especially children. I will be very thankful if there are families who accept this kind of thing when it happens. It is not easy for the family to just send you to a place where you can get initiated. Instead they will do a cleansing ceremony for you to get the spirits out” (Female, FGD1, Isangoma/Inyanga).*

Once they were qualified healers, THPs were perceived with scepticism by other community members. For example, one THP said that as THPs they had foresight to identify individuals who had the calling to become THPs even before those people knew or recognised the calling. They were able to connect to the ancestral spirits of those individuals. They said that they were able to reveal this kind of information because of their ability to know and foresee things before they could happen, however the open disclosure of such information was perceived by the community as inappropriate.

*“When you are at church you can see a child or an older person and see that they have the ancestral spirits. You can feel their spirit. When we are talking amongst ourselves we say, that person is a Sangoma, but has not yet realized it, we laugh. Others will say that just because I am a Sangoma now I am saying everyone is a Sangoma. I tell them that I can feel another Sangoma’s spirit” (Female, FGD1, Isangoma/Inyanga)*

THPs who were not openly practicing and had kept their calling undisclosed in the community were also referred to and described as THPs by other healers.

*“ehhhh my child there are many, I will give you a wrong figure if you want to know the numbers, there are many of them, others don’t want to be known. Others they have hidden themselves, they don’t want to be known” (Female, FGD1, Isangoma/Inyanga)*

### Typology and role of traditional healers

Throughout the group discussions, three main categories of THPs were identified and raised by participants: 1) *Isangoma* (diviner); 2) *Inyanga* (one who focuses on traditional medical remedies) and 3) *Umthandazi* (faith healer).

Below, we describe different THP types, the processes involved in assuming a particular type of THP and mode specific roles carried by different types of healers.

1. *Isangoma (Diviner)*

*Isangoma* (diviner) was reported as the most common type of THP that existed in this study community. Participants identified three critical stages in a healers’ pathway to becoming a *Sangoma* (diviner): (1) *Ubizo* (the calling or gift) which was sent by *Amadlozi* (ancestors); (2) *ukuthwasa* (training or initiation) which involved being initiated and trained into healing; and lastly (3) *Ukuphothula* (completion), a period at the end of training, which also marked the beginning of a *Sangoma’s* (diviner’s) practice of healing. The stepwise process of becoming a *Sangoma* (diviner) was described by one THP:

*“…..for a Sangoma, someone [an ancestor] will come to you in a dream and tell you that you have to go and be initiated. After that you will run away crying until you reach another [a sangoma who will facilitate the process of training] Sangomas house. When you get there, that Sangoma [trainer] will welcome you. She will talk to her own ancestors and let them know that you are there to be initiated. The (ancestor) who came to you in a dream will also be with you [spiritually] to teach you how to do ukuhlola (divination). That Sangoma will do everything until the end. At the end she will take you back home, accompanied by other Sangomas. After that you will be able to talk and connect to ancestors, you will be able to tell people their problems” (Female, FGD1, Isangoma/Inyanga)*

Also mentioned in the above narrative is *ukuthwasa* (training or initiation) which takes place through a trainer (*a diviner*) who is chosen by the ancestors. The above narrative further illustrates that the ancestors give absolute direction, from choosing the person they want to bestow the gift upon, to the trainer, the process of training, completion as well as informing the practice.

Moreover, it demonstrates that *Sangomas* (diviners) do not all subscribe to the same method of calling. While the calling for some was revealed through dreams, for others it was revealed through illness or a combination of different methods marked by physical, emotional or psychological illness (ancestral illness).

In the role of *Isangoma* (diviner), the THP used *Ukuhlola* (divination) to determine the cause and source of illness, and offer an explanation to patients. Additionally, *Isangoma* used *Umuthi* (medicines) in their treatments. It was indicated that for *Sangomas* (diviners) to do their work, they operated by means of ancestral spirits, referred to as *umndiki* and *umndawe* (ancestral spirits).

*“….It is because of the spirits that possess us, they are called Umndiki or Umndawe” (FGD3, Female, Isangoma/Inyanga)*

2. *Umthandazi (Faith healer)*

THPs in group discussions identified religion and spirituality as playing a crucial role in the initiation and practice of *Umthandazi* (faith healer). One participant said that a messenger, in the form of a spirit called *Isithunywa* (God-sent messenger)*,* would come to the person whom the calling is bestowed upon and that person will then be able to connect to the ancestral and spiritual realms.

*“….when the spirits arrive it is unexpected as he was explaining. We usually say it is bursting [surprising and shocking] just because we do not expect it to happen, especially in front of other people. I was born in a home where we were praying a lot under the church of Nkonyane Zion” (Female, FGD1, Umthandazi/Inyanga)*

Participants indicated *that Amadlozi* (ancestors) are awakened and elevated in a relationship which is focused on healing. God works together with *Amadlozi* (ancestors) to bring protection and restore wellbeing. The spirit of God and *Amadlozi* (ancestors) were mentioned as subjects of respect and honour, which go hand in hand and cannot be separated. THPs pointed out that *Amadlozi* (ancestors) and *Isithunywa* (God-sent messengers), sometimes referred to as *Imimoya* (Godly spirits) worked within *Umthandazi* (faith healer) to reveal things that were hidden, and it was through this process that *Umthandazi* (faith healer) could learn about other people’s problems and be able to help people. The ancestral spirits and *Isithunywa* (God sent-messengers) gave guidance in the practice of *Umthandazi* (faith healer).

*“When the spirits rise [start working] it means that the person starts speaking in a way that he does not know. He does not know how he got to speak like that. That is how the spirits start to operate in him” (Male, FGD1, Umthandazi/Inyanga)*

In the role of *Umthandazi* (faith healer)*,* prayer and water are the main methods of healing, but minerals such as ash and salt could also be used in the process of healing.

*“When I pray as Umthandazi, I use water and salt. I cook those little things (salts). You pray by using water, ash and incense those kinds of things”. (Female, FGD1, Umthandazi/Inyanga)*

3. *Inyanga (One who focuses on traditional medical remedies)*

THPs reported that *Inyanga* (herbalist) *was someone who went* through a period of intensive apprenticeship to learn about using plants, herbs in traditional medicinal remedies and connecting with *Amadlozi* (ancestors). The apprenticeship was taken with an existing *Inyanga* (herbalist). While *Inyanga* (herbalists) depended more on the knowledge gathered during the apprenticeship, there was still a need to connect to *Amadlozi* (ancestors) to activate the healing ability.

*“When a person comes to me and enters at Endumbeni [consultation room], I then consult with the ancestors and come back to mix umuthi [medicine] for a patient” (Female, FDG4, Traditional healer)*

*Inyanga’s* (herbalist’s) role did not only involve prescribing traditional medicines to patients, it was reported that *Inyanga* (herbalists) also performed curative rituals for their patients. One *inyanga* (herbalist) healer said that in their work they often asked patients to perform certain rituals or undergo certain practices in the process of healing.

*“Yes the child will drink the medicine outside (household premises) and also take the bath outside. We do not use a bath tub. I just pour the mixture over the child’s head and the mother will run her hands through the child’s body. We all walk back to the house and we do not look back. When we look back, it means that we are looking at the thing that we were removing from this child and it will come back again to trouble the child” (Female, FGD4, Inyanga)*

4. *Multiple categories and roles*

Six out of nine THPs in this study assumed more than one THP category. Three were both *Isangoma/Inyanga* (diviner/herbalist), two were *Umthandazi/Inyanga* (faith healer, herbalist*)* and one was *Isangoma/Umthandazi* (diviner/faith healer)*.* They indicated that one healer could be called into multiple THP categories. As a result, that healer could practice or get trained in different types of healing, for example as both *Isangoma* (diviner) and *Inyanga* (herbalist), thus be able to carry out multiple traditional healing roles.

*“…..here is someone among us, we know that she is a leader in her church but she is also a Sangoma. It has not been even five years since she started being a Sangoma. She has been helping people within the church (as Umthandazi). But she is now a Sangoma. She went for initiation and stayed in someone (trainer) else’s home [to train as a Sangoma] even though she has ISithunywa” (Female, FGD1, Isangoma/Inyanga)*

When one undertook multiple categories, the calling, training and even practice did not run concurrently. It was said that when a healer practiced between different types, *Amadlozi* (ancestors) determined how the healing was to be undertaken.

*“When a person comes to me for assistance, I can tell if I need to use ISithunywa (as Umthandazi) or if I need to use the herbs (as Inyanga). That is how a person becomes both Umthandazi and Inyanga or Sangoma”. (Female, FGD1, Umthandazi/Inyanga)*

THPs said that they also held other roles in the community and lived normal lives. However, those roles had to be acknowledged and guided by the ancestors. In instances where roles such as being a wife or a parent were in conflict with the calling, the calling would always prevail, and events would conspire to ensure this occurred. Other roles could not be in conflict with the traditional healing call. This is shown in the quotation below, where one THP describes how her partner did not pay *lobola* (bride wealth) for her because her parents were refusing to accept her calling and send her for initiation. The THP believed that her partner was used by the ancestors to punish her and her parents.

*“…..I stayed like that, I was never initiated until I reached a stage of having a boyfriend and having children. That person [partner] did not pay any lobola [bridewealth] for me; we just stayed in a relationship [cohabited]. I had four children, one of my daughters even got married but my partner did not pay lobola for me because the ancestors were angry when I did not take up the calling, this was their way of communicating with me and my family” (Female, FGD1, Isangoma/Inyanga)*

### Ancillary roles of traditional health practitioners

These ancillary roles were not specific to certain types of THP’s, but were generally assumed and described as part of a generic THP role, regardless of type. All the healers reported one or more additional roles they undertook within the different categories of healing.

THPs reported that in addition to providing explanations for causation and source of illness they also provided protection and counselling to their patients as directed by *Amadlozi* (ancestors)*.* THPs indicated that they managed difficulties such as reconciling relationships and they provided protection (through mediation with *ancestors* and/or God and through providing treatment) to individual people or families.

#### Counselling

THPs reported that they were able to reconcile marriages or relationships that had gone wrong.

*“When you are quarrelling and the woman does not even want to have sex with the husband, the other one doesn’t want the other one [estrangement between partners]. If one of them comes to me and tells me that he wants to make things right in their marriage, I will tell him to come back with his partner so that I can give them umuthi together” (Female, FGD 3, Isangoma)*

Participants reflected that the role of THP was a pressured one in which patient expectations were often high, even in cases where the THP would not consider the patient’s problem to be one which fell within the scope of traditional healing.

*“…..they ask you to give them amagobongo [a mixture of traditional herbs used for cleansing the body or treating a particular illness]. Like P5 was saying, I also agree with her. People come and ask for amagobongo when you can see that what they have has nothing to do with idlozi (ancestor)” (Female, FGD3, Inyanga)*

When they realised that they were unable to assist their patients, they advised them to go to the local clinic and they made referrals for them. All but one of the THPs showed a willingness to engage with other medical practices and had been trained by the African Medical and Research Foundation (AMREF) around managing and responding to TB and HIV-infected patients, symptoms of HIV, referring patients to Western biomedical institutions. Those who were trained said that the training strengthened their understanding of HIV and TB, which untrained THPs often lacked.

*“We were given referral cards. We use them to refer patients which we cannot help spiritually or with traditional medicine. We write on this referral card all the symptoms that they tell us and ask them to take it to the clinic” (Female, FGD3, Isangoma/Inyanga)*

#### Protection

One THP said that the mixtures of *umuthi* (traditional medicine) that they use are also helpful in providing safety and security within the homes.

*“I use it to sprinkle the yard to chase away bad spirits and thieves or imikhovu [a spirit of a dead person that is used in witchcraft]. You see there have never been any thieves in my house. Maybe they are afraid of my boys, I don’t know. But I believe that it is this intelezi [African plant that people use to cleanse themselves or rid themselves of evil spirits in their homes or places of businesses] that is helping me. I mix the herbs with water and I sprinkle in the yard and in the kraal. I sprinkle it all over” (Female, FGD4, Isangoma/Inyanga)*

THPs stated that they were able to protect people from *isinyama* [dark omens]. Removing *isinyama* helped individuals to carry out their daily duties without interferences that may be caused by witchcraft or ancestral wrath.

*“Sometimes when people go to the clinic, they will not be taken care of because they have isinyama (dark omens) that is following them. People will not even want to look at them and nurses will not want anything to do with them. So when such people come to me, the first thing I do is to cleanse them, to remove isinyama so that people can be able to look at them and they must be attractive to people” (Female, FGD3, Inyanga)*

#### Mediation

THPs indicated two ways of communicating with the ancestors. One way was when the ancestors communicated with the living using metaphors (dreams and visions) or significant others or events to communicate.

*“While I was seeing people’s secrets in the church, then someone came to me at night [in a dream] when I was sleeping. That person said “I can see that you are able to see people’s secrets”, but he said he wanted me to smear ibomvu (traditional red clay). I asked [again] if they wanted me to smear ibomvu”. (Female, FGD1, Umthandazi/Inyanga)*

A second way was achieved by invoking the spirits and by performing rituals. Rituals included slaughtering of animals and making offerings to the ancestors.

*“…..someone from the family gave me a cow to slaughter to appease the ancestors”. (Female, FGD1, Isangoma/Inyanga)*

All the THPs described their working space as sanctified and it was where they could communicate with the ancestors.

*“…..When they are at endumbeni [Healers consultation room], I do ukuhlola (divination) using my bones. Once I see what their problems are….the bones will show me what is troubling the person” (Female, FGD4, Isangoma)*

Table 3 summarises the mode specific process and roles of THPs and generic ancillary roles that were not specific to THP type.

**Table 3:** Characteristics of different kinds of traditional healers

Typology of traditional health practitioners			
	ISangoma	UMthandazi	INyanga
Process to become THP	. Called by *Amadlozi*. Trained by an expert through the guidance and instruction of *Amadlozi*	. Invoked by the spirit of I*sithunywa* and *Amadlozi*	. Individual choice to become an *Inyanga*
			. Apprenticeship with an expert
Role	. Divination Contact and communication with A*madlozi* (those of healer and those of a patient)	. Prayer Provides holy water and other mineral substances such as ash and salt to facilitate healing	. Treatment, which includes herbs, plants and traditional rituals
	. Traditional rituals		
Ancillary roles			
• Counselling • Protecting • Mediating			

## Discussion

This exploratory study offers insight into the role of THPs, the process into becoming healers and how THPs perceive their healing practice in relation to the different categories described in this study as well as other previous studies in the literature [16, 40]. Our results show that a majority of THPs in this study occupy multiple categories of healing, that is, they practice across different healing types. Our study also demonstrates that THPs’ role performance includes services that go beyond the uses of herbs for physical illnesses or divination. THPs serve roles which include, but are not limited to, custodians of the traditional African religion and customs, educators about culture, counsellors, mediators and social protectors. All the healers put emphasis on their relationship and connection to the ancestral and spiritual worlds, and said that who they were and how they executed healing was from the guidance of the ancestors.

In occupying multiple categories, THPs carried out roles across different types, therefore maximising the services they offered to their patients, without their patients having to travel from one THP to another. Conversely, in the literature, THPs roles are treated as mutually exclusive, they include: herbalists (*izinyanga* or *amaxhwele*), diviners (*izangoma*, *umthandazi* or *amagqirha*), traditional surgeons (*iingcibi*) who mainly do circumcisions, and traditional birth attendants (*ababelethisi* or *abazalisi*) [16, 41, 42].

However, Rautenbach [40] notes that the differences that exist between the different types of THPs have often been vague as a result of biased and conflicting accounts of academic writers, and by the differences that exist between the various tribal groups in South Africa. Rautenbach states that the continuous labelling of THPs as a collective, i.e. “traditional healers” does not capture the generic and distinct roles of THPs shown in this study and says this is a result of the colonial powers and structures which have played a role in changing the setting of traditional healing in South Africa [40].

THPs described additional services that they offered to their patients. These services were generic across healing types and were related to their knowledge of protective herbs and rituals; their knowledge of handling and managing witchcraft or ancestral wrath; and being mediators between the living and the ancestors. Within the *Zulu* tradition, when family members died, they are said to remain connected to the living [10]. Therefore, *Amadlozi* are regarded to be a part of family functioning, and they are remembered by the living.

*Amadlozi* are included in family decision-making processes and are called upon to provide protection and prosperity [43]. They watch over their living relatives, guiding them if they adhere to laws of living, reprimanding them if they break the laws [44]. These laws include observing life-cycle rituals that are due to the ancestors, such as *Imbeleko* (a ritual performed to introduce a baby to the ancestors and the family at large), initiation rituals, marriage rituals and rituals performed after death of a family member, and before and after the burial [10]. Being mediums to the ancestral/spiritual worlds was described as a unique feature in their role-performance. According to Ross [43], people gain access to the ancestral and spiritual world through mediums (healers). In this regard, THPs occupy a role of facilitating communication between the living and *Amadlozi* in the form of divination and rituals [45].

THPs said that some of their patients’ problems were not within the scope of traditional healing. In order for their patients to receive appropriate heath care, most THPs said they referred them to western biomedical facilities. Several studies have explored the willingness of THPs to work alongside biomedical practitioners and they have shown that THPs express a readiness to learn beyond their own healing system [25, 46, 47]. At this stage, talks about the “practical” regulation of traditional healing are vague, leaving THPs pressured by the demands of fitting into the broader health system and needing more support and less isolation from authorities concerned with health care in South Africa.

The study demonstrates that assuming a THP role is often met with difficulty both from the individual in which the role is bestowed upon and from others, specifically family members. THPs described not understanding what was going on to them when the calling manifested. The same feeling was shared by their family members. These views are also shown in a study that was conducted in South Africa among Bapedi THPs [24]. However, Sodi et al. [16] provide a contrasting view. In their study, they found that communities were happy and they accepted and supported THPs in their roles. Being a THP was seen as being like a king [16]. This study was however conducted among the Venda and Tsonga tribes. In the current study, THPs said that if they adhered to the calling, respected and followed the guidance from their ancestors, they could assume other roles and live “normal” lives which were not related to being a THP.

What is common in the literature about the identity of THPs is that it is directly influenced and linked to the ancestors [16, 22, 24]. In a commentary, Cumes [48] states that THPs conduct their practice with humbleness and constantly acknowledge that the source of their healing ability is the ancestors. Acknowledgement implies not just the source of the calling, but also the understanding that the gift they possess requires receiving and accepting spiritual guidance from the ancestral world, without which, they cannot be identified as THPs [22]. The focal point of traditional healing is within the ancestral and spiritual realms [49]. THPs said that becoming a healer was inevitable. These sentiments were particularly shared by *Sangomas* and *Abathandazi* (plural for Umthandazi) and they have been acknowledged in other studies [8, 14, 48, 50]. Further, when those chosen resist the calling gift, they suffer punishment in the form of persistent illness (physical, psychological or socially), thus at some point they need to answer the calling by submitting to the role they have been assigned [50]. However, it was not clear how *Inyanga* is chosen or elected into the practice.

This study provides an initial insight into the roles of traditional healers, more clarity on the nuances of becoming *iNyanga* and *uMthandaz*i are needed. THPs in this study did not provide a clear understanding about how *Inyanga* is initiated into this role. It was said that it required intensive training about medicinal herbs as well as learning to connect with the ancestors in order to facilitate the process of training. It was not clear whether *Inyanga* could practice without using the ancestral connection, and if so how this is to be done. THPs described in detail how the calling to become I*sangoma* manifests, however narratives around the calling to become *Inyanga* and *Umthandazi* were not as detailed. We cannot speculate if this was due to the fact that *Isangoma* was the most prominent type of healer in this community, resulting in more information about the role of *Isangoma*. The study demonstrates the roles of THPs in spiritual enhancement, an aspect omitted in the Traditional Health Practitioners Act (No. 35 of 2004), thus illustrating a broader aspect of traditional healing which goes beyond focusing on the physical body. Findings from this study can be used to guide the ministry of health in South Africa as it moves towards formally integrating traditional healing and medicine into the public health system.

### Limitations

The results of this study should be interpreted within their methodological context. The study uses data collected as part of a larger TasP trial (ANRS 12249), therefore questions did not only focus on the roles and different identities of traditional healers, but also on other aspects of TasP such as HIV testing, ART and early initiation of ART. The study used purposive sampling, and participants were selected based on judgement about which ones will be most useful or representative [51]. The study also used snowball sampling, which meant that THPs were referred because they were known by the initial four who were purposefully sampled, excluding other THPs in the community. We acknowledge that the sampling techniques used in this study may have introduced some bias, as THPs were not enrolled from a standardised register of THPs in the Hlabisa sub-district. Even though other studies [22, 52] demonstrate that generally more practicing THPs are female, we do not know the full population and gender composition of THPs in this area and so cannot tell how typical our sample is of all THPs.

## Conclusion

In conclusion, the study indicates that THPs have generic and mode specific roles. The mode specific roles are influenced by the processes by which they become healers. There are specific types of methods and practices used by specific healers, which are not similar across healing types. Generic roles and methods include those that are similar across THP types. The labelling of THPs as “traditional healers” does not capture the generic and distinct roles of THPs. However, we learnt that it has been commonly used in the literature to refer to THPs in a general sense. THPs who occupied multiple roles did not perceive themselves, and were not perceived by other healers, as superior or inferior to healers who practiced exclusively as one type of healer. It is unclear whether a hierarchy among different types of healers exists or not: Further research is needed to explore this issue.

## Abbreviations

AIDS: Acquired immune deficiency syndrome
AMREF: African Medical and Research Foundation
ART: Antiretroviral therapy
BREC: Biomedical research ethics committee
CAB: Community advisory board
CEU: Community engagement unit
FGD: Focused group discussion
HIV: Human immunodeficiency virus
HPCSA: Health professions council of South Africa
KZN: KwaZulu-Natal
PMTCT: Prevention of mother to child transmission
TasP: Treatment as prevention
TB: Tuberculosis
THPs: Traditional health practitioners
WHO: World Health Organisation
